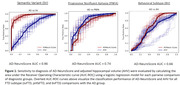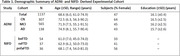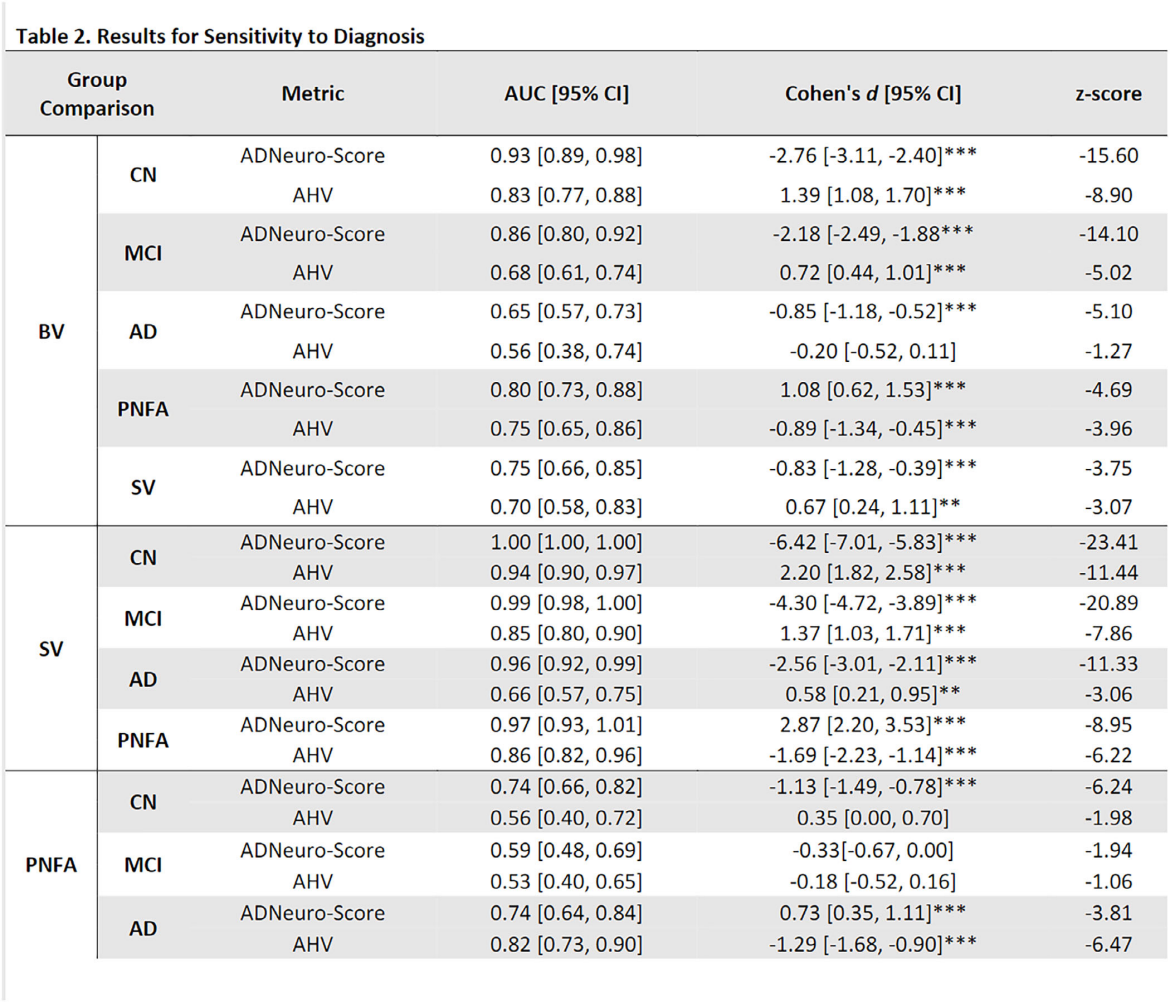# Preliminary Validation of a Structural Magnetic Resonance Imaging Metric for Differential Diagnosis of Frontotemporal Lobe Dementias

**DOI:** 10.1002/alz.091935

**Published:** 2025-01-09

**Authors:** Gavin T Kress, Emily S. Popa, Paul M. Thompson, Susan Y. Bookheimer, Sophia I Thomopoulos, Christopher RK Ching, Hong Zheng, David A. Merrill, Cyrus A. Raji, Stella E. Panos, Prabha Siddarth, Jennifer E. Bramen

**Affiliations:** ^1^ Pacific Brain Health Center, Pacific Neuroscience Institute and Foundation, Santa Monica, CA USA; ^2^ The Icahn School of Medicine at Mount Sinai, New York, NY USA; ^3^ Imaging Genetics Center, Mark and Mary Stevens Neuroimaging and Informatics Institute, Keck School of Medicine, University of Southern California, Marina del Rey, CA USA; ^4^ Pacific Brain Health Center, Pacific Neuroscience Institute Foundation, Santa Monica, CA USA; ^5^ David Geffen School of Medicine at University of California Los Angeles, Los Angeles, CA USA; ^6^ Imaging Genetics Center, Mark and Mary Stevens Neuroimaging & Informatics Institute, Keck School of Medicine, University of Southern California, Marina del Rey, CA USA; ^7^ Mallinckrodt Institute of Radiology, Washington University in St. Louis, St. Louis, MO USA; ^8^ Saint John's Cancer Institute at Providence Saint John's Health Center, Santa Monica, CA USA

## Abstract

**Background:**

AD‐NeuroScore is a validated metric that summarizes Alzheimer’s disease (AD)‐specific atrophy and can detect AD early, benchmark disease severity, predict and monitor AD progression, and can aid in testing the efficacy of therapeutic interventions using a single number (Kress, 2023). It meets criteria for translatability by using clinically available regional brain volumes as input (Ahdidan, 2015; Cavedo, 2022) and having patient and model‐level interpretability (Pinto, 2022). Also, features can be reviewed by a neuroradiologist (Larson, 2019). Scores are harmonized for intracranial volume, age, sex, and scanner model, improving workflows for longitudinal patient tracking. The objective of this study is to examine whether AD‐NeuroScore can be used in the differential diagnosis of AD and FTD dementia subtypes.

**Method:**

Cognitively normal (CN) individuals and patients with a mild cognitive impairment (MCI), AD, behavioral variant (bv)‐FTD, semantic variant (sv)‐FTD, and progressive nonfluent aphasia (pnfa)‐FTD diagnoses were drawn from the ADNI and NIFD cohort studies. Eighty‐four cortical and subcortical regional volumes were estimated from T1‐weighted MR images using FreeSurfer. AD‐NeuroScore uses a modified Euclidean‐inspired distance function to calculate differences between individuals and a CN template based on regional brain volumes associated with cognitive decline (Kress, 2023). Validation used an experimental cohort (Table 1) and tested sensitivity to differentiating CN, MCI, and AD from FTD‐subtypes (bv, sv, and pnfa) using pairwise t‐tests and an alpha=0.001, Bonferroni corrected. Area under the curve (AUC) was calculated. Results were benchmarked against adjusted hippocampal volume (AHV), an NIA‐AA diagnostic biomarker for AD (Jack, 2018), which was also harmonized for intracranial volume, age, sex, and scanner.

**Result:**

Both AD‐NeuroScore and AHV distinguished between AD and svFTD and pnfaFTD variants. AD‐NeuroScore better differentiated svFTD from AD than AHV (Table 2; Figure 1). AHV performed as well or qualitatively better than AD‐NeuroScore at distinguishing AD from pnfaFTD. Neither AD‐NeuroScore nor AHV differentiated AD and bv‐FTD.

**Conclusion:**

AD‐NeuroScore differentiated between AD, svFTD, and pnfaFTD and overall, performed equivalently to or better than AHV. Performance and utility in longitudinal tracking may be improved by creating FTD variant scores using with a focus on regions specific to FTD subtypes.